# Impact of pancreatic ductal occlusion on postoperative outcomes in pancreatic head cancer patients undergoing neoadjuvant therapy

**DOI:** 10.1007/s00535-024-02125-8

**Published:** 2024-06-20

**Authors:** Yoshifumi Hidaka, Shiroh Tanoue, Takuro Ayukawa, Koji Takumi, Hirotsugu Noguchi, Michiyo Higashi, Tetsuya Idichi, Yota Kawasaki, Hiroshi Kurahara, Yuko Mataki, Takao Ohtsuka, Chihaya Koriyama

**Affiliations:** 1 Department of Epidemiology and Preventive Medicine, Graduate School of Medical and Dental Science, Kagoshima University, 8-35-1 Sakuragaoka, Kagoshima, 890-8544 Japan; 2 Department of Digestive Surgery, Graduate School of Medical and Dental Science, Kagoshima University, 8-35-1 Sakuragaoka, Kagoshima, 890-8544 Japan; 3 Department of Radiology, Graduate School of Medical and Dental Science, Kagoshima University, 8-35-1 Sakuragaoka, Kagoshima, 890-8544 Japan; 4 Department of Pathology, Graduate School of Medical and Dental Science, Kagoshima University, 8-35-1 Sakuragaoka, Kagoshima, 890-8544 Japan

**Keywords:** Pancreatic ductal occlusion, Pancreatic head cancer, Pancreatic exocrine insufficiency, Malnutrition, Neoadjuvant therapy

## Abstract

**Background:**

Pancreatic ductal occlusion can accompany pancreatic head cancer, leading to pancreatic exocrine insufficiency (PEI) and adverse effects on nutritional status and postoperative outcomes. We investigated its impact on nutritional status, body composition, and postoperative outcomes in patients with pancreatic head cancer undergoing neoadjuvant therapy (NAT).

**Methods:**

We analyzed 136 patients with pancreatic head cancer who underwent NAT prior to intended pancreaticoduodenectomy (PD) between 2015 and 2022. Nutritional and anthropometric indices (body mass index [BMI], albumin, prognostic nutritional index [PNI], Glasgow prognostic score, psoas muscle index, subcutaneous adipose tissue index [SATI], and visceral adipose tissue index) and postoperative outcomes were compared between the occlusion (*n* = 78) and non-occlusion (*n* = 58) groups, in which 61 and 44 patients, respectively, ultimately underwent PD.

**Results:**

The occlusion group showed significantly lower post-NAT BMI, PNI, and SATI (*p* = 0.011, 0.005, and 0.015, respectively) in the PD cohort. The occlusion group showed significantly larger main pancreatic duct, smaller pancreatic parenchyma, and greater duct–parenchymal ratio (*p* < 0.001), and these morphological parameters significantly correlating with post-NAT nutritional and anthropometric indices. Postoperative 3-year survival and recurrence-free survival (RFS) rates were significantly poorer (*p* = 0.004 and 0.013) with pancreatic ductal occlusion, also identified as an independent postoperative risk factor for overall survival (hazard ratio [HR]: 2.31, 95% confidence interval [CI] 1.08–4.94, *p* = 0.030) and RFS (HR: 2.03, 95% CI 1.10–3.72, *p* = 0.023), in multivariate analysis.

**Conclusions:**

Pancreatic ductal occlusion may be linked to poorer postoperative outcomes due to PEI-related malnutrition.

## Introduction

Pancreatic cancer has an extremely poor prognosis, with the worst 5-year survival rate (12%) among cancers covered by United States statistics [1]. Pancreatectomy remains the only curative option; however, it is highly invasive, entails risks of postoperative pancreatic fistula (POPF) [2], delayed gastric emptying (DGE) [3], and post-pancreatectomy hemorrhage (PPH) [4], and is associated with a high (79%) early postoperative recurrence rate [5]. Postoperative outcomes could be improved by identifying novel prognostic indicators to inform clinical decision making.

Tumor size and differentiation, lymph node metastasis, and resection margin are known prognosticators of pancreatic cancer [6, 7]; however, the relevant data cannot be obtained preoperatively. Consequently, preoperative clinical assessment items have been investigated as potential prognosticators, including prognostic nutritional index (PNI) [8], Glasgow prognostic score (GPS) [9], psoas muscle index (PMI) [10], and subcutaneous adipose tissue (SAT), and visceral adipose tissue (VAT) [11].

Neoadjuvant treatment (NAT) has recently become the mainstream treatment for pancreatic cancer and is reportedly associated with prolonged survival [12–14]. However, NAT tends to extend the waiting period prior to surgery, and is reportedly associated with impaired nutritional status [15, 16] and body composition [17–19]. Furthermore, NAT-associated impaired body composition is a potential negative prognosticator in patients with pancreatic cancer [19]. Malnutrition should thus be assessed during NAT, using blood biochemical and imaging examinations.

Pancreatic head cancer can have a poorer prognosis when the tumor causes ductal occlusion resulting in exocrine insufficiency (PEI)-related malnutrition [20, 21]; however, the impact of pancreatic ductal occlusion has not previously been characterized in patients with this cancer.

Accordingly, in this study, we aimed to investigate the effects of pancreatic ductal occlusion on nutritional status, body composition, and postoperative outcomes in patients with pancreatic head cancer who underwent NAT prior to intended pancreaticoduodenectomy (PD).

## Methods

This retrospective observational study was conducted in compliance with the Declaration of Helsinki, and was approved by the Institutional Review Bord of Kagoshima University Hospital. The requirement for obtaining informed consent was waived owing to the retrospective nature of the study; however, the content of the study was publicly disclosed on the Kagoshima University Hospital homepage, enabling the participants or their designated representatives to opt out of participation.

### Patients

We identified 173 consecutive patients diagnosed with pancreatic head cancer who underwent NAT prior to intended PD at Kagoshima University Hospital, between January 1, 2015 and December 31, 2022. The analysis set comprised 136 patients after excluding cases corresponding to absence of pre-NAT computed tomography (CT) images (n = 5), preoperative ductal stent placement (n = 5), pancreatic enzyme replacement therapy (PERT) (n = 24), or variance in clinical course (n = 3). All clinical data were obtained from the electronic medical records of Kagoshima University Hospital and its affiliated institutions.

### Evaluation and definition of pancreatic ductal occlusion

**Fig. 1:**
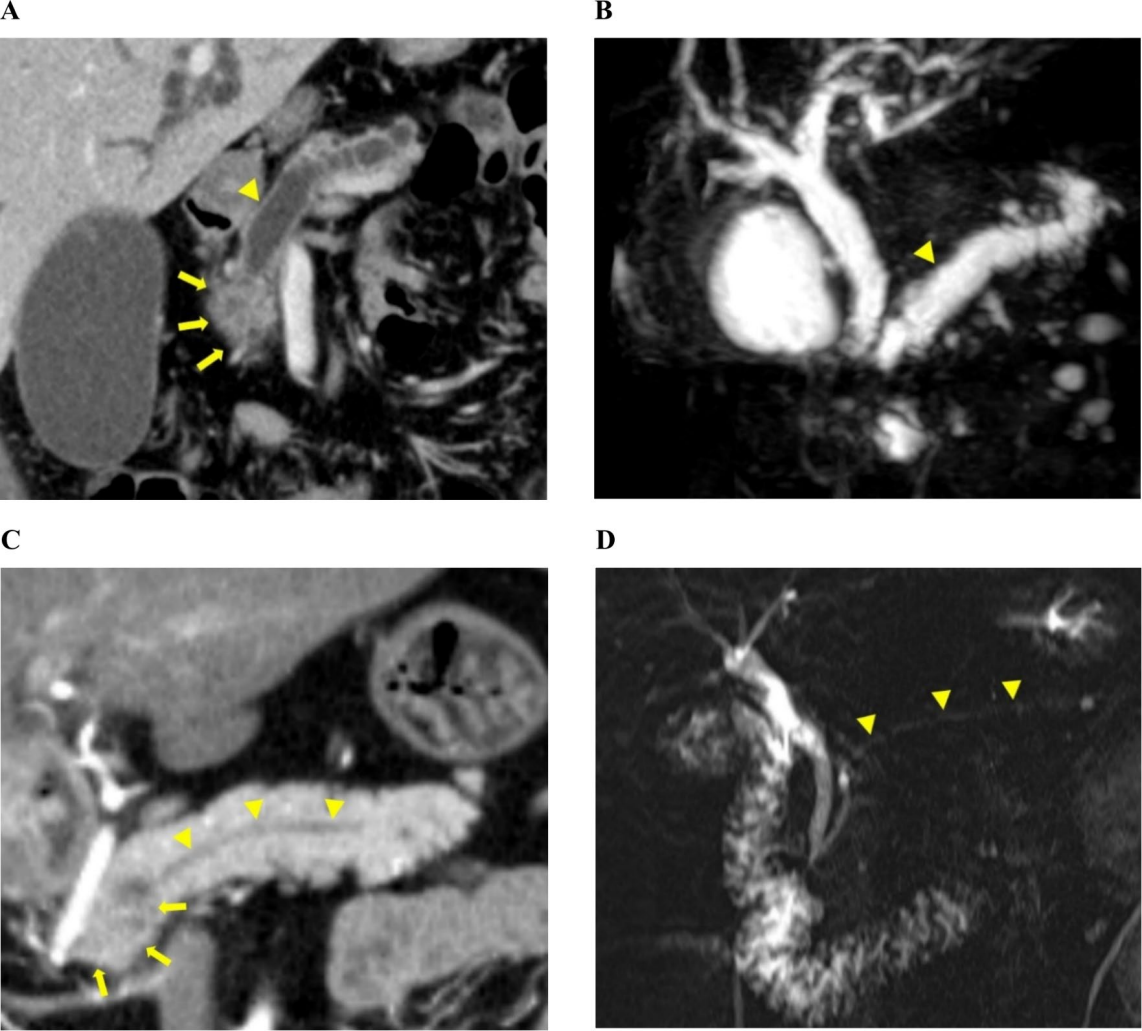
Representative images of cases with and without pancreatic ductal occlusion. **A** and** B** CT (**A**) and MRCP images (**B**) of a 69-year-old man with pancreatic ductal occlusion showing pancreatic cancer (arrows) and a dilated main pancreatic duct (arrowhead). **C** and** D**, CT (**C**) and MRCP images (**D**) of a 73-year-old man without pancreatic ductal occlusion showing pancreatic cancer (arrows) and an undilated main pancreatic duct (arrowhead). *CT* computed tomography, *MRCP* magnetic resonance cholangiopancreatography

Pancreatic ducts were evaluated for occlusion (Fig. 1A, B) or non-occlusion (Fig. 1C, D) by a radiologist (TA, KT) and the principal investigator (YH) using pre-NAT CT images. Magnetic resonance cholangiopancreatography images were referenced when available.

Pancreatic ductal occlusion was defined as tumor obstruction of both the main and accessory ducts and a dilated upstream main pancreatic duct (MPD) ≥ 3 mm, which was set as the cutoff referring to a previous report [22]. The MPD was measured on cross-sectional CT images of the presumed transection line of the pancreas near the portal vein.

### Neoadjuvant treatment and adjuvant chemotherapy

The NAT regimens in this study were as follows: (i) gemcitabine plus tegafur/gimeracil/oteracil (S-1) therapy (GS therapy) (*n* = 43) [23]; (ii) nab-paclitaxel plus gemcitabine therapy (GN therapy) (*n* = 50) [24]; (iii) chemoradiotherapy (CRT) with or without additional GS or GN therapy (CRT) (*n* = 35); and (iv) other treatments (*n* = 8). CRT included external-beam radiation with concurrent S-1 administration. Other regimens included a modified combination therapy of folinic acid, fluorouracil, irinotecan, and oxaliplatin (mFOLFIRINOX) (*n* = 1) [25]; any two chemotherapy regimens from S-1 monotherapy, GS, GN, and mFOLFIRINOX therapy (*n* = 4); S-1 plus proton therapy (*n* = 2); and radiation-alone therapy (*n* = 1). The content, intensity, and cycles of NAT were established in accordance with standard protocols, but were adjusted when necessary, by the attending physician based on the patient's condition and treatment efficacy.

In this study, adjuvant chemotherapy involved a 6-month regimen of S-1 monotherapy as the standard protocol [26]. Patients who experienced adverse reactions to S-1 then received gemcitabine monotherapy (*n* = 6) [26] or GN therapy (*n* = 1). Twenty patients did not receive adjuvant chemotherapy.

### Clinicopathological data

The following data were collected: age, sex, resectability (based on the National Comprehensive Cancer Network guidelines version 2. 2021), NAT regimen, completion of NAT and PD, and the interval (days) from the initiation of NAT to surgery. In addition, we collected data on complications and determined from a history of treatment or preoperative blood biochemical examination findings (diabetes: hemoglobin A1c ≥ 6.5%; chronic kidney disease: estimated glomerular filtration rate < 60 mL/min/1.73 m^2^), pre- and post-NAT carbohydrate antigen 19–9 (CA 19–9) levels, the provision of preoperative biliary drainage, hematologic adverse events after NAT (≥ grade 3 based on Common Terminology Criteria for Adverse Event version 5.0), NAT treatment effect (based on Response Evaluation Criteria in Solid Tumors version 1.1), tumor-node-metastasis stage (based on the Union for International Cancer Control 8th edition), pathological tumor size, histological type and grade, Evans’ criteria [27], resection margin, operation time, operative blood loss, postoperative hospital stay, postoperative complications (Clavien–Dindo classification ≥ grade IIIa; POPF ≥ grade B [2]; DGE ≥ grade B [3]; and PPH ≥ grade B [4]), and provision of adjuvant chemotherapy and postoperative PERT.

### Nutritional assessment

Body mass index (BMI) was obtained at pre- and post-NAT assessments. Albumin concentration (g/dL), lymphocyte count (/mm^3^), and C-reactive protein (CRP) (mg/dL) were obtained at pre- and post-NAT blood biochemical assessments. Pre-NAT BMI and blood biochemical data were obtained 18.0 days (interquartile range [IQR] 2.0–26.0 days) and 24.0 days (IQR 17.0–33.0 days) before NAT initiation, respectively. Post-NAT BMI and blood biochemical data were obtained 2.0 days (IQR 1.0–3.0 days) and 18.0 days (IQR 13.0–27.0 days) before PD, respectively.

Onodera’s PNI (10 × albumin g/dL + 0.005 × lymphocyte /mm^3^) [8] and GPS (GPS score = 2: both CRP > 1.0 mg/dL and albumin < 3.5 g/dL; GPS score = 1: CRP > 1.0 mg/dL or albumin < 3.5 g/dL; GPS score = 0: neither CRP > 1.0 mg/dL nor albumin < 3.5 g/dL) [9] were evaluated as described previously, using pre- and post-NAT blood biochemical data.

### Image analysis

Pre-NAT CT images were obtained 32.0 days (IQR 21.0–42.0 days) before NAT initiation, and post-NAT CT images were obtained 24.0 days (IQR 14.0–41.0 days) before PD. Pancreatic morphology was evaluated using pre-NAT cross-sectional CT images. The MPD (mm) and pancreas (mm) were measured on the presumed transection line of the pancreas near the portal vein. Pancreatic parenchyma (mm) was calculated as follows:

Pancreatic parenchyma (mm) = size of the pancreas (mm) – size of the MPD (mm).

In addition, the ratio of the MPD to parenchymal thickness was calculated as follows [28]:

Duct–parenchymal ratio = size of the MPD (mm)/size of the pancreas (mm).

To evaluate muscle mass, L3-level cross-sectional CT images obtained pre- and post-NAT were used. The cross-sectional area of the bilateral psoas muscles (cm^2^) was measured by manual tracing, and the PMI was calculated as follows [10]:

PMI = Area of bilateral psoas muscle/height^2^ (cm^2^/m^2^).

To evaluate adipose tissue content, Dicom images corresponding to the L3-level cross-sectional CT images obtained pre- and post-NAT were analyzed automatically using SYNAPSE VINCENT software (Fujifilm Medical Co., Ltd., Tokyo, Japan). Tissue HU thresholds were set as follows: −190 to −30 HU for SAT and −150 to −50 HU for VAT [29]. SAT (cm^2^) and VAT (cm^2^) were divided by the square of the height to calculate the SAT index (SATI) and VAT index (VATI), respectively, as follows:

SATI = SAT/height^2^ (cm^2^/m^2^).

VATI = VAT/height^2^ (cm^2^/m^2^).

### Statistical analyses

Data are presented as percentage or median and IQR values. Differences between groups were evaluated using the χ^2^ test, Fisher’s exact test, or Mann–Whitney *U* test. A two-sided *p* < 0.05 was regarded as statistically significant. Relationships between pancreatic morphology and nutritional and anthropometric indices were analyzed with Spearman’s rank correlation coefficient (rs). The Kaplan–Meier method was used to evaluate the 3-year survival and recurrence-free survival (RFS) rates after PD, and comparisons between groups were performed using the log-rank test. Univariate and multivariate analyses were performed using the Cox proportional-hazard model to identify potential prognostic factors influencing overall survival (OS) and RFS after PD. In the multivariate model, age, sex, and pancreatic ductal occlusion were selected as the clinically relevant variables, while resectability, pathological TNM stage and tumor size were selected as risk adjusting variables clinically influencing the survival analysis of this study. Stata version 18 (Stata Corp LLC, College Station, TX, USA) was used for all analyses.

## Results

### Clinicopathological characteristics

A total of 136 patients were assigned to the occlusion (*n* = 78) or non-occlusion (*n* = 58) group, and 105 of these patients ultimately underwent PD (occlusion group: *n* = 61; non-occlusion group: *n* = 44) (Table 1). For these 105 patients (PD cohort) showed, the histological tumor classifications were as follows: adenocarcinoma (*n* = 58), adenosquamous carcinoma (*n* = 1), and residual carcinoma (*n* = 2) in the occlusion group; and adenocarcinoma (*n* = 42), adenosquamous carcinoma (*n* = 1), and high-grade pancreatic intraepithelial neoplasia (*n* = 1) in the non-occlusion group.

**Table 1:** Clinicopathological characteristics

	Occlusion		Non-occlusion		*p* value
	No. of Pts/MD	*N*(%) or Median(IQR)	No. of Pts/MD	*N*(%) or Median(IQR)	
Age (years)	78/0	69.0 (65.0, 74.0)	58/0	68.5 (63.0, 74.0)	0.358
Sex (male)	78/0	46 (59)	58/0	33 (57)	0.808
Resectability (R/BR/UR)	78/0	46 (59)/30 (38)/2 (3)	58/0	46 (79)/11 (19)/1 (2)	**0.026**
NAT regimen (GS/GN/CRT/others)	78/0	23 (30)/25 (32)/26 (33)/4 (5)	58/0	20 (34)/25 (43)/9 (16)/4 (7)	0.121
NAT completion, yes	78/0	66 (85)	58/0	54 (93)	0.129
Pancreaticoduodenectomy completion, yes	78/0	61 (78)	58/0	44 (76)	0.747
Days from NAT initiation to Surgery	61/0	126.0 (98.0, 198.0)	44/0	95.5 (75.0, 127.5)	**0.002**
MPD (mm)	78/0	5.2 (4.0, 7.5)	58/0	2.0 (1.3, 2.5)	** < 0.001**
Pancreatic parenchyma (mm)	78/0	7.1 (5.3, 9.9)	58/0	11.1 (8.7, 14.5)	** < 0.001**
Duct–parenchymal ratio	78/0	0.4 (0.3, 0.6)	58/0	0.1 (0.1, 0.2)	** < 0.001**
Diabetes, present	76/2	30 (39)	56/2	18 (32)	0.387
Chronic kidney disease, present	78/0	10 (13)	58/0	6 (10)	0.658
Pre-NAT CA 19–9 (U/mL)	77/1	87.8 (22.8, 237.7)	57/1	42.2 (8.1, 132.0)	0.059
Post-NAT CA 19–9 (U/mL)	75/3	22.3 (10.1, 53.5)	55/3	15.0 (6.7, 42.4)	0.153
Preoperative biliary drainage, yes	78/0	50 (64)	58/0	33 (57)	0.394
Hematologic NAT AE (≥ grade 3), present	75/3	40 (53)	54/4	30 (56)	0.803
RECIST (PR/SD/PD)	76/2	16 (21)/55 (72)/5 (7)	57/1	18 (32)/36 (63)/3 (5)	0.393
Pre-NAT TNM stage (I/II/III)	78/0	62 (79)/14(18)/2 (3)	58/0	43 (74)/12 (21)/3 (5)	0.657
Post-NAT TNM stage (I/II/III/IV)	76/2	61 (80)/11 (15)/3 (4)/1 (1)	57/1	45(79)/7(12)/3 (5)/2 (4)	0.840
Pathological TNM stage (0/I/II/III/IV)	61/0	0/24 (39)/29 (48)/7 (11)/1 (2)	44/0	1 (2)/21 (48)/16 (36)/6 (14)/0	0.523
Pathological tumor size (mm)	61/0	25.0 (20.0, 30.0)	43/1	21.0 (15.0, 25.0)	0.077
Pathological N stage (N0/N1/N2)	61/0	25 (41)/28 (46)/8 (13)	44/0	22 (50)/16 (36)/6 (14)	0.599
Histological type (Adenocarcinoma/Others)	61/0	58 (95)/3 (5)	44/0	42 (95)/2 (5)	1.000
Histological grade (well/mod/por)	52/9	28 (54)/22 (42)/2 (4)	42/2	24 (57)/18 (43)/0	0.646
Evans’ criteria (I/IIa/IIb/III)	54/7	41 (76)/9 (16)/2 (4)/2 (4)	38/6	30 (79)/5 (13)/3 (8)/0	0.624
Resection margin (R0/R1)	60/1	57 (95)/3 (5)	44/0	43 (98)/1 (2)	0.636
Operating time (min)	61/0	488.0 (410.0, 557.0)	44/0	427.5 (375.0, 539.0)	0.087
Operative blood loss (mL)	61/0	605.0 (414.0, 1090.0)	44/0	598.5 (346.0, 1002.5)	0.610
Postoperative hospital stay (days)	61/0	15.0 (12.0, 18.0)	44/0	18.5 (14.0, 22.0)	**0.009**
Clavien–Dindo classification (≥ grade IIIa), present	61/0	7 (11)	44/0	12 (27)	**0.038**
POPF (≥ grade B), present	61/0	1 (2)	44/0	12 (27)	** < 0.001**
DGE (≥ grade B), present	61/0	8 (13)	44/0	9 (20)	0.314
PPH (≥ grade B), present	61/0	1 (2)	44/0	1 (2)	1.000
Adjuvant chemotherapy, yes	61/0	48 (79)	44/0	37 (84)	0.487
Postoperative PERT, yes	59/2	51 (86)	44/0	41 (93)	0.345

Bold values denote statistical significance at *p* < 0.05

*Pts* patients, *MD* missing data, *IQR* interquartile range, *R* resectable, *BR* borderline resectable, *UR* unresectable, *NAT* neoadjuvant therapy, *GS* gemcitabine plus S-1, *GN* gemcitabine plus nab-paclitaxel, *CRT* chemoradiotherapy, *MPD* main pancreatic duct, *CA19-9* carbohydrate antigen 19–9, *AE* adverse event, *RECIST* Response Evaluation Criteria in Solid Tumors, *PR* partial response, *SD* stable disease, *PD* progressive disease, *TNM* tumor-node-metastasis, *mod* moderately differentiated, *por* poorly differentiated, *POPF* postoperative pancreatic fistula, *DGE* delayed gastric emptying, *PPH* post-pancreatectomy hemorrhage, *PERT* pancreatic enzyme replacement therapy

The occlusion group showed significantly larger MPD, smaller pancreatic parenchyma, and greater duct–parenchymal ratio (*p* < 0.001). Patients in the occlusion group also experienced fewer postoperative complications, with significantly lower incidences of Clavien–Dindo classification ≥ grade IIIa complications (*p* = 0.038) and POPF (*p* < 0.001).

### Nutritional and anthropometric indices at the pre- and post-NAT assessments

At pre-NAT, ductal occlusion was associated with significantly lower BMI and VATI for all patients (*p* = 0.015 and 0.028), and significantly lower BMI (*p* = 0.048) in the PD cohort (Table 2).

**Table 2:** Nutritional and anthropometric indices at the pre-NAT assessment

	Occlusion		Non-occlusion		*p* value
	No. of Pts/MD	Median(IQR)	No. of Pts/MD	Median(IQR)	
All Pts (*n* = 136)					
BMI (kg/m^2)^	77/1	21.3 (19.3, 23.4)	58/0	22.5 (20.9, 24.9)	**0.015**
Albumin (g/dL)	74/4	4.0 (3.6, 4.3)	56/2	3.9 (3.7, 4.2)	0.590
PNI	70/8	47.0 (43.7, 49.2)	51/7	45.7 (42.9, 51.0)	0.739
GPS	70/8	0 (0, 0)	53/5	0 (0, 0)	0.720
PMI (cm^2^/m^2^)	78/0	4.5 (3.7, 5.4)	58/0	4.9 (3.8, 5.8)	0.188
SATI (cm^2^/m^2^)	77/1	32.3 (20.8, 46.7)	57/1	34.9 (26.1, 54.3)	0.132
VATI (cm^2^/m^2^)	77/1	34.6 (18.5, 52.2)	57/1	42.2 (23.6, 64.5)	**0.028**
PD cohort (*n* = 105)					
BMI (kg/m^2)^	60/1	21.4 (19.3, 23.8)	44/0	23.0 (20.6, 24.9)	**0.048**
Albumin (g/dL)	58/3	4.1 (3.7, 4.3)	42/2	3.9 (3.7, 4.2)	0.654
PNI	54/7	47.2 (43.1, 49.2)	38/6	46.1 (44.1, 51.6)	0.924
GPS	54/7	0 (0, 0)	40/4	0 (0, 0)	0.421
PMI (cm^2^/m^2^)	61/0	4.6 (3.8, 5.4)	44/0	4.9 (3.7, 5.9)	0.706
SATI (cm^2^/m^2^)	60/1	31.7 (21.3, 46.2)	43/1	34.9 (26.2, 57.7)	0.124
VATI (cm^2^/m^2^)	60/1	35.8 (16.1, 56.3)	43/1	42.2 (20.8, 64.5)	0.115

Bold values denote statistical significance at *p* < 0.05

*NAT* neoadjuvant therapy, *Pts* patients, *MD* missing data, *IQR* interquartile range, *BMI* body mass index, *PNI* prognostic nutritional index, *GPS* Glasgow prognostic score, *PMI* psoas muscle index, *SATI* subcutaneous adipose tissue index, *VATI* visceral adipose tissue index, *PD* pancreaticoduodenectomy

At post-NAT, ductal occlusion was associated with significantly lower BMI, PNI, SATI, and VATI in all patients (*p* = 0.002, 0.023, 0.007, and 0.014, respectively), and significantly lower BMI, PNI, and SATI in the PD cohort (*p* = 0.011, 0.05, and 0.015, respectively) (Table 3).

**Table 3:** Nutritional and anthropometric indices at the post-NAT assessment

	Occlusion		Non-occlusion		*p* value
	No. of Pts/MD	Median(IQR)	No. of Pts/MD	Median(IQR)	
All Pts (*n* = 136)					
BMI (kg/m^2)^	78/0	20.6 (19.0, 23.1)	58/0	23.3 (20.1, 25.2)	**0.002**
Albumin (g/dL)	74/4	3.7 (3.4, 4.0)	56/2	3.9 (3.5, 4.1)	0.208
PNI	74/4	42.8 (39.3, 48.1)	55/3	45.9 (42.4, 48.1)	**0.023**
GPS	73/5	0 (0, 1)	56/2	0 (0, 1)	0.363
PMI (cm^2^/m^2^)	76/2	4.5 (3.5, 5.3)	57/1	4.6 (3.8, 5.6)	0.141
SATI (cm^2^/m^2^)	74/4	26.5 (14.9, 36.7)	57/1	32.7 (24.3, 47.2)	**0.007**
VATI (cm^2^/m^2^)	74/4	27.4 (14.3, 42.0)	57/1	38.9 (21.4, 58.9)	**0.014**
PD cohort (*n* = 105)					
BMI (kg/m^2)^	61/0	21.0 (19.3, 23.7)	44/0	23.4 (20.4, 25.4)	**0.011**
Albumin (g/dL)	60/1	3.7 (3.4, 4.1)	44/0	4.0 (3.6, 4.1)	0.097
PNI	60/1	42.8 (39.6, 48.1)	43/1	46.4 (44.0, 48.6)	**0.005**
GPS	60/1	0 (0, 1)	44/0	0 (0, 0)	0.065
PMI (cm^2^/m^2^)	60/1	4.5 (3.8, 5.6)	43/1	4.6 (3.6, 5.9)	0.556
SATI (cm^2^/m^2^)	59/2	27.6 (15.6, 37.2)	43/1	35.5 (24.3, 46.8)	**0.015**
VATI (cm^2^/m^2^)	59/2	30.6 (14.1, 42.1)	43/1	38.9 (20.7, 58.9)	0.072

Bold values denote statistical significance at *p* < 0.05

*NAT* neoadjuvant therapy, *Pts* patients, *MD* missing data, *IQR* interquartile range, *BMI* body mass index, *PNI* prognostic nutritional index, *GPS* Glasgow prognostic score, *PMI* psoas muscle index, *SATI* subcutaneous adipose tissue index, *VATI* visceral adipose tissue index, *PD* pancreaticoduodenectomy

### Correlations between pancreatic morphology and post-NAT nutritional and anthropometric indices

All patients and the PD cohort showed significant correlations between pancreatic morphology and nutritional and anthropometric indices; between MPD and BMI, PNI, and SATI (inversely); between parenchyma and BMI, PMI, SATI, and VATI; and between duct–parenchymal ratio and BMI, PMI, SATI, and VATI (Table 4).

**Table 4:** Relation between pancreatic morphology and post-NAT nutritional and anthropometric indices

	All Patients (*n* = 136)			PD cohort (*n* = 105)		
	No. of Pts/MD	rs	*p* value	No. of Pts/MD	rs	*p* value
MPD						
BMI (kg/m^2)^	136/0	−0.283	** < 0.001**	105/0	−0.241	**0.013**
Albumin (g/dL)	130/6	−0.101	0.255	104/1	−0.146	0.139
PNI	129/7	−0.216	**0.014**	103/2	−0.299	**0.002**
GPS	129/7	0.058	0.512	104/1	0.178	0.071
PMI (cm^2^/m^2^)	133/3	−0.157	0.071	103/2	−0.086	0.390
SATI (cm^2^/m^2^)	131/5	−0.324	** < 0.001**	102/3	−0.314	**0.001**
VATI (cm^2^/m^2^)	131/5	−0.248	**0.004**	102/3	−0.170	0.087
Parenchyma						
BMI (kg/m^2)^	136/0	0.398	** < 0.001**	105/0	0.372	** < 0.001**
Albumin (g/dL)	130/6	−0.062	0.481	104/1	−0.024	0.806
PNI	129/7	0.032	0.716	103/2	0.075	0.449
GPS	129/7	−0.005	0.954	104/1	−0.038	0.703
PMI (cm^2^/m^2^)	133/3	0.333	** < 0.001**	103/2	0.312	**0.001**
SATI (cm^2^/m^2^)	131/5	0.364	** < 0.001**	102/3	0.334	** < 0.001**
VATI (cm^2^/m^2^)	131/5	0.360	** < 0.001**	102/3	0.324	**0.001**
DP ratio						
BMI (kg/m^2)^	136/0	−0.377	** < 0.001**	105/0	−0.333	** < 0.001**
Albumin (g/dL)	130/6	−0.027	0.763	104/1	−0.077	0.438
PNI	129/7	−0.160	0.070	103/2	−0.243	**0.013**
GPS	129/7	0.031	0.727	104/1	0.125	0.205
PMI (cm^2^/m^2^)	133/3	−0.260	**0.003**	103/2	−0.199	**0.044**
SATI (cm^2^/m^2^)	131/5	−0.394	** < 0.001**	102/3	−0.362	** < 0.001**
VATI (cm^2^/m^2^)	131/5	−0.341	** < 0.001**	102/3	−0.259	**0.009**

Bold values denote statistical significance at *p* < 0.05

*NAT* neoadjuvant therapy, *PD* pancreaticoduodenectomy, *Pts* patients, *MD* missing data, *MPD* main pancreatic duct, *BMI* body mass index, *PNI* prognostic nutritional index, *GPS* Glasgow prognostic score, *PMI* psoas muscle index, *SATI* subcutaneous adipose tissue index, *VATI* visceral adipose tissue index, *DP* duct–parenchymal

### Survival analysis after PD

**Fig. 2:**
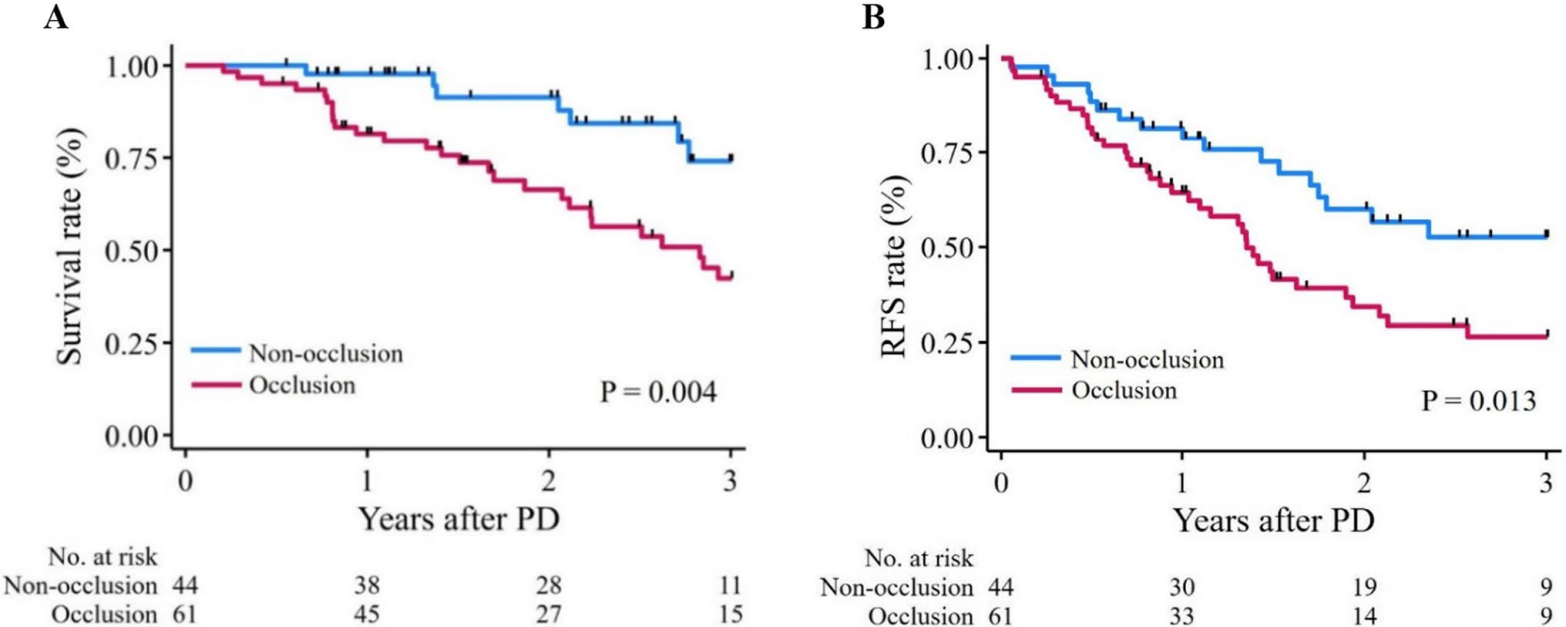
3-year survival and RFS rates after PD. **A** and** B** survival rates (**A**) and RFS rates (**B**). *RFS* recurrence-free survival, *PD* pancreaticoduodenectomy

Median post-PD follow-up was 749.0 days (IQR, 399.0–1091.0 days) for all patients and 792.5 days (IQR, 412.0–1132.0 days) for censored patients. During the follow-up period, 41 patients died and 59 patients experienced recurrence. The occlusion group showed significantly lower postoperative 3-year survival and RFS rates (*p* = 0.004 and 0.013) (Fig. 2A, B).

Risk factors for survival were evaluated with univariate and multivariate analyses (Table 5). The former revealed pancreatic morphology (ductal occlusion and MPD dilation), post-NAT nutritional indices (albumin and GPS), pathological TNM and N stages, and histological grade as significant postoperative risk factors for both OS and RFS. The latter revealed pancreatic ductal occlusion as an independent postoperative risk factor for OS (hazard ratio [HR], 2.31; 95% confidence interval [CI] 1.08–4.94, *p* = 0.030) and RFS (HR, 2.03; 95% CI 1.10–3.72; *p* = 0.023).

**Table 5:** Risk factors for OS and RFS after PD

	No. of Pts/MD	Univariate analysis			Multivariate analysis		
		HR	95% CI	*p* value	HR	95% CI	*p* value
OS							
Age (≥ 69 years: median)	57/0	0.79	0.42–1.45	0.441	1.15	0.57–2.31	0.705
Sex (Male)	63/0	1.32	0.70–2.50	0.393	1.19	0.60–2.36	0.611
Pancreatic ductal occlusion (yes)	61/0	2.65	1.30–5.43	**0.008**	2.31	1.08–4.94	**0.030**
MPD (≥ 3.0 mm)	64/0	2.71	1.29–5.71	**0.009**			
Pancreatic parenchyma (< 9.3 mm: median)	48/0	1.51	0.81–2.81	0.191			
Duct–parenchymal ratio (≥ 0.3: median)	46/0	2.04	1.08–3.83	**0.027**			
Post-NAT albumin (< 3.8 g/dL: median)	47/1	3.41	1.75–6.63	** < 0.001**			
Post-NAT PNI (< 44.0: median)	46/2	2.03	1.08–3.81	**0.027**			
Post-NAT GPS (1 + 2)	32/1	2.78	1.45–5.34	**0.002**			
Post-NAT SATI (< 28.0 cm^2^/m^2^: median)	49/3	1.24	0.66–2.34	0.496			
Post-NAT VATI (< 31.5 cm^2^/m^2^: median)	49/3	1.20	0.63–2.26	0.583			
Pre-NAT CA19-9 (≥ 64.0 U/mL: median)	49/2	1.59	0.84–3.01	0.151			
Resectability (BR + UR)	31/0	1.64	0.85–3.16	0.137	1.64	0.82–3.29	0.163
Pathological TNM stage (II + III + IV)	59/0	2.24	1.16–4.33	**0.017**	2.47	1.18–5.18	**0.016**
Pathological tumor size (≥ 25 mm: median)	53/1	1.37	0.73–2.57	0.321	1.01	0.52–1.98	0.976
Pathological N stage (N1 + N2)	58/0	2.16	1.13–4.12	**0.020**			
Histological grade (mod + por)	42/11	2.10	1.06–4.17	**0.035**			
RFS							
Age (≥ 69 years: median)	57/0	0.48	0.28–0.80	**0.005**	0.56	0.33–0.97	**0.037**
Sex (Male)	63/0	1.43	0.84–2.44	0.188	1.21	0.69–2.13	0.512
Pancreatic ductal occlusion (yes)	61/0	1.95	1.13–3.38	**0.017**	2.03	1.10–3.72	**0.023**
MPD (≥ 3.0 mm)	64/0	2.27	1.27–4.03	**0.005**			
Pancreatic parenchyma (< 9.3 mm: median)	48/0	1.20	0.71–2.02	0.489			
Duct–parenchymal ratio (≥ 0.3: median)	46/0	1.40	0.84–2.35	0.200			
Post-NAT albumin (< 3.8 g/dL: median)	47/1	2.07	1.22–3.50	**0.007**			
Post-NAT PNI (< 44.0: median)	46/2	1.52	0.90–2.55	0.114			
Post-NAT GPS (1 + 2)	32/1	2.02	1.16–3.53	**0.013**			
Post-NAT SATI (< 28.0 cm^2^/m^2^: median)	49/3	1.14	0.67–1.94	0.631			
Post-NAT VATI (< 31.5 cm^2^/m^2^: median)	49/3	0.99	0.59–1.67	0.968			
Pre-NAT CA19-9 (≥ 64.0 U/mL: median)	49/2	1.87	1.10–3.18	**0.020**			
Resectability (BR + UR)	31/0	0.91	0.51–1.63	0.762	0.85	0.47–1.55	0.604
Pathological TNM stage (II + III + IV)	59/0	1.92	1.12–3.31	**0.018**	1.73	0.98–3.05	0.059
Pathological tumor size (≥ 25 mm: median)	53/1	1.24	0.74–2.06	0.417	0.81	0.45–1.44	0.470
Pathological N stage (N1 + N2)	58/0	1.85	1.08–3.17	**0.025**			
Histological grade (mod + por)	42/11	1.81	1.05–3.12	**0.032**			

Bold values denote statistical significance at *p* < 0.05

*OS* overall survival, *RFS* recurrence-free survival, *PD* pancreaticoduodenectomy, *Pts* patients, *MD* missing data, *HR* hazard ratio, *CI* confidence interval, *MPD* main pancreatic duct, *NAT* neoadjuvant therapy, *PNI* prognostic nutritional index, *GPS* Glasgow prognostic score, *SATI* subcutaneous adipose tissue index, *VATI* visceral adipose tissue index, *CA 19–9* carbohydrate antigen 19–9, *BR* borderline resectable, *UR* unresectable, *TNM* tumor-node-metastasis, *mod* moderately differentiated, *por* poorly differentiated

## Discussion

Our results indicate that pancreatic ductal occlusion is associated with impaired post-NAT nutritional status and adipose tissue content in patients with pancreatic head cancer undergoing NAT, and that it serves as a postoperative risk factor for PD. To our knowledge, this is the first report to describe the influence of pancreatic ductal occlusion on the treatment of pancreatic head cancer.

In other studies, NAT has been linked with impaired nutritional status and body composition, and significantly decreased PNI [15, 16], BMI and subcutaneous fat [17], visceral fat [18], and skeletal muscle and visceral and subcutaneous fat [19] in patients with pancreatic cancer. Reduced skeletal muscle and visceral fat during NAT may indicate poor prognosis [19]. In this study, the occlusion group showed significantly poorer post-NAT nutritional and anthropometric indices. This group also showed significantly larger MPD, smaller parenchyma, and greater D–P ratio and these morphological parameters significantly correlated with post-NAT nutritional and anthropometric indices. Furthermore, poorer postoperative 3-year survival and RFS rates were associated with occlusion. Given that pancreatic morphology and post-NAT nutritional indices were significant postoperative risk factors for both OS and RFS, PEI-related malnutrition during NAT may negatively affect prognosis of pancreatic head cancer patients. Pancreatic ductal occlusion was also an independent postoperative risk factor for OS and RFS, suggesting its potential as prognosticator mediated by PEI-related malnutrition.

Patients with pancreatic head cancer may develop preoperative PEI due to pancreatic ductal occlusion [20, 21], and preoperative pancreatic atrophy may lead to postoperative PEI. Preoperative CT evaluation of pancreatic morphology can thus be utilized to access PEI-related malnutrition. In patients with pancreatic cancer, the pancreatic exocrine secretory capacity reportedly begins to decline when more than 60% of the pancreatic duct is obstructed, with an exponentially greater decline when the obstruction is located closer to the ductal orifice [30]. The incidence of PEI is 44% following PD and 20% following distal pancreatectomy (DP), with corresponding postoperative prevalence rates of 74% (range, 36–100%) and 67–80% [31]. Pylorus-preserving PD reportedly yielded a significantly greater decrease in pancreatic exocrine capacity than DP, as well as a significantly greater MPD, thinner pancreatic parenchyma, and larger duct-to-parenchymal ratio in preoperative CT evaluations of pancreatic morphology [32]. In another study, patients judged to have a hard pancreas during surgery showed significantly larger MPD and smaller pancreatic parenchyma on preoperative CT images, as well as a significantly larger fibrosis ratio and smaller lobular ratio histologically, and a significantly lower incidence of POPF [33]. These reports are consistent with our findings that the occlusion group showed significantly larger MPD, smaller pancreatic parenchyma, and greater duct–parenchymal ratio in pre-NAT CT examinations and a significantly lower incidence of POPF. Furthermore, our study showed pancreatic morphology significantly correlated with nutritional and anthropometric indices, suggesting that PEI should be suspected when pancreatic-head-cancer-related ductal occlusion with concomitant CT-detectable morphological changes are observed. One study reported only 1.9% of patients with pancreatic cancer underwent PEI testing [34]. This low testing rate may be explained by difficulties in conducting relevant diagnostic tests (e.g., fecal esterase-1, coefficient of fat absorption [CFA], and ^13^C-mixed triglyceride breath test). CT assessments of pancreatic ductal occlusion and concomitant morphological changes thus have potential utility as practical indicators of PEI in pancreatic head cancer patients.

Pancreatic-ductal-occlusion-related PEI should be addressed during the initial phase of pancreatic head cancer management. PERT is the standard therapy for PEI; however, the effect of this therapy has primarily been investigated in unresectable pancreatic cancer or post-pancreatectomy. In one such study, patients with unresectable pancreatic cancer and suspected pancreatic ductal obstruction showed significantly improved body weight (BW) after PERT [35]. In another study, PERT significantly improved the post-pancreatectomy CFA, BW, and BMI [36]. PERT is also an independent prognosticator following PD (HR: 0.75, 95% CI 0.57–0.99, *p* = 0.046), significantly improving survival in patients with MPD ≥ 3 mm (HR: 0.64, 95% CI 0.47–0.89, *p* = 0.006), but not in those with MPD < 3 mm (HR: 1.01, 95% CI 0.66–1.55, *p* = 0.970) [37]. Endoscopic pancreatic stenting may also be an effective treatment option for pancreatic ductal occlusion, since it significantly delayed the development of PEI in chronic pancreatitis patients with MPD stenosis [38]. Effective treatment of PEI due to pancreatic ductal occlusion can improve nutritional status and survival outcomes; thus, evaluation of pancreatic ductal occlusion is crucial in pancreatic head cancer cases.

This study has several limitations. First, the results may have been influenced by heterogeneity such as that arising from variations in NAT regimens and duration. Second, the evaluation of the pancreatic ductal occlusion was not blinded. Third, we cannot rule out the possibility that patients assigned to the non-occlusion pre-NAT CT group developed occlusion while undergoing NAT. Fourth, some missing data were identified, but they showed no skewed intergroup distribution; thus, statistical analysis was performed with the missing data included.

In conclusion, pancreatic ductal occlusion may be linked to poorer postoperative outcomes through PEI-related malnutrition.
